# Complete Genome Sequence of an Avian Paramyxovirus Representative of Putative New Serotype 13

**DOI:** 10.1128/genomeA.00729-16

**Published:** 2016-07-28

**Authors:** Iryna Goraichuk, Poonam Sharma, Borys Stegniy, Denys Muzyka, Mary J. Pantin-Jackwood, Anton Gerilovych, Olexii Solodiankin, Vitaliy Bolotin, Patti J. Miller, Kiril M. Dimitrov, Claudio L. Afonso

**Affiliations:** a National Scientific Center Institute of Experimental and Clinical Veterinary Medicine, Kharkiv, Ukraine; b Exotic and Emerging Avian Viral Disease Research Unit, Southeast Poultry Research Laboratory, U.S. National Poultry Research Center, ARS, USDA, Athens, Georgia, USA

## Abstract

Here, we report the complete genome sequence of a virus of a putative new serotype of avian paramyxovirus (APMV). The virus was isolated from a white-fronted goose in Ukraine in 2011 and designated white-fronted goose/Ukraine/Askania-Nova/48-15-02/2011. The genomic characterization of the isolate suggests that it represents the novel avian paramyxovirus group APMV 13.

## GENOME ANNOUNCEMENT

Avian paramyxoviruses (APMVs) belong to the genus *Avulavirus* of the family *Paramyxoviridae*, order *Mononegavirales* (1). There are 12 confirmed (APMV-1 to -12) serotypes in the genus (2, 3). Virulent strains of APMV-1 cause Newcastle disease (ND), one of the most devastating diseases in poultry. The rest of the serotypes have been associated with mild infections in poultry, wild and domestic waterfowl, passerines, psittacines, and columbids, among other species (4–11). Three of the recently accepted serotypes (APMV-10 to -12) were isolated from Rockhopper penguin, Common snipe, and Eurasian wigeon, respectively (12–14). Viruses from the genus *Avulavirus* have a genome size of approximately 15 kb, consisting of a negative-sense single-stranded RNA, with six genes encoding up to eight different proteins.

In 2011, active surveillance of wild birds for avian influenza (AI) virus and APMV was conducted in the Azov-Black Sea region of Ukraine. A hemagglutinating agent that did not cross-react with AI virus antisera was isolated from fecal samples collected from a white-fronted goose (*Anser albifrons*) in the Kherson region. The isolate weakly cross-reacted with antisera against APMV-1 and APMV-7 but did not show any cross-reactivity with the other APMV serotypes. The intracerebral pathogenicity index (ICPI) test (15) resulted in a value of 0.34, which is indicative of a virus of low virulence for chickens.

Complete genome sequencing of the virus was conducted at the Southeast Poultry Research Laboratory, Athens, GA. Viral RNA was isolated from allantoic fluid using the QIAamp RNA viral minikit (Qiagen, USA). The complete genome sequence was obtained using next-generation sequencing employing the Nextera XT DNA library preparation kit (Illumina, San Diego, CA). The distribution size and concentration of the prepared libraries were checked on a Bioanalyzer 2100, using the Agilent high-sensitivity DNA kit (Agilent Technologies, Germany), and Qubit, using the double-stranded DNA (dsDNA) HS assay kit (Life Technologies, USA). Paired-end sequencing was performed on an Illumina MiSeq instrument using the 500-cycle MiSeq reagent kit version 2 (Illumina). Sequence data were assembled using MIRA version 3.4.0 (16) within a customized workflow on the Galaxy platform (17).

Sequence analysis revealed that the complete genome length of the isolated strain designated white-fronted goose/Ukraine/Askania-Nova/48-15-02/2011 is 16,146 nucleotides (nt) (in accordance with the “rule of six”). The viral genome contained six transcriptional units (3′-NP-P-M-F-HN-L-5′) of 1,482 nt, 1,194 nt, 1,101 nt, 1,638 nt, 1,740 nt, and 6,600 nt in length, respectively. The genome sequence showed 97% nucleotide identity to that of goose/Kazakhstan/5751/2013 (accession no. KU646513) that was recently deposited in GenBank and designated APMV-13 (18). However, the genome of white-fronted goose/Ukraine/Askania-Nova/48-15-02/2011 is 150 nt longer than the one of the virus isolated in Kazakhstan. The 5ˊ and 3ˊ ends of the genome reported here were confirmed as described previously (19). The fusion protein gene of the white-fronted goose/Ukraine/Askania-Nova/48-15-02/2011 isolate had 98% nucleotide identity to APMV/goose/Shimane/67/2000, isolated in Japan from goose fecal samples and also reported previously as APMV-13 (20). Phylogenetic evaluation of the predicted amino acid sequences of the viral proteins revealed that among the rest of the serotypes, APMV-12 is most closely related to the newly characterized virus (63.7 to 75.1% amino acid identity).

### Nucleotide sequence accession number.

The complete genome sequence of APMV/white-fronted goose/Askania-Nova/48-15-02/11 has been deposited in GenBank under the accession no. KX119151.
